# A continuing clinical education course to maintain clinical competencies and foster new clinical knowledge during the graduate school years of MD-PhD training

**DOI:** 10.1017/cts.2023.694

**Published:** 2023-12-14

**Authors:** Graham D. Cochrane, Shima D. Anwar, Alice N. Weaver, Stephanie N. Brosius, Catherine H. Poholek, Heather Allen, Randy L. Seay, Robin G. Lorenz, Gregory A. Payne, William M. Geisler

**Affiliations:** 1 Heersink School of Medicine, University of Alabama at Birmingham, Birmingham, AL, USA; 2 Department of Pediatrics, Boston Children’s Hospital, Harvard Medical School, Boston, MA, USA; 3 Division of Medical Oncology, University of Colorado Anschutz Medical Campus, Aurora, CO, USA; 4 Division of Neurology, Children’s Hospital of Philadelphia, Perelman School of Medicine at the University of Pennsylvania, Philadelphia, PA, USA; 5 Department of Pediatrics, Division of Pediatric Rheumatology, UPMC Children’s Hospital of Pittsburgh, Pittsburgh, PA, USA; 6 Division of Hospital Medicine, Stritch School of Medicine, Loyola University Medical Center, Maywood, IL, USA; 7 Department of Research Pathology, Genentech, South San Francisco, CA, USA

**Keywords:** MD-PhD, clinical, education, knowledge, survey

## Abstract

**Introduction:**

Most students in MD-PhD programs take a leave of absence from medical school to complete PhD training, which promotes a natural loss of clinical skills and knowledge and could negatively impact a student’s long-term clinical knowledge. To address this concern, clinical refresher courses in the final year of PhD training have traditionally been used; however, effectiveness of such courses versus a longitudinal clinical course spanning all PhD training years is unclear.

**Methods:**

The University of Alabama at Birmingham MD-PhD Program implemented a comprehensive continuing clinical education (CCE) course spanning PhD training years that features three course components: (1) clinical skills; (2) clinical knowledge; and (3) specialty exposure activities. To evaluate course effectiveness, data from an anonymous student survey completed at the end of each semester were analyzed.

**Results:**

Five hundred and ninety-seven surveys were completed by MD-PhD students from fall 2014 to 2022. Survey responses indicated that the majority of students found the course helpful to: maintain clinical skills and knowledge (544/597, 91% and 559/597, 94%; respectively), gain exposure to clinical specialties (568/597, 95%), and prepare them for responsibilities during clinical clerkships. During semesters following lockdowns from the COVID-19 pandemic, there were significant drops in students’ perceived preparedness.

**Conclusions:**

Positive student survey feedback and improved preparedness to return to clinic after development of the course suggests the CCE course is a useful approach to maintain clinical knowledge during research training.

## Introduction

MD-PhD programs are integrated dual-degree programs designed to accelerate development of physician-scientists who will advance human health using tools gained through rigorous research and clinical training. Traditionally, MD-PhD training lasts approximately 8 years with three consecutive stages: medical science education, PhD training, and clinical training (i.e., clerkships and electives) [1,2]. Attrition rates within MD-PhD programs are estimated to be between 10% and 15% [1,2], which may be related to students’ prolonged duration of training (leading to stress and short-term income loss), difficulty transitioning between training stages, and lack of institutional support [1–4]. Given the decline of the physician-scientist workforce [1,2], innovative programs to mitigate these factors are urgently needed.

The transition into clinical training is one of the most challenging aspects of medical school, but this re-entry is particularly salient for MD-PhD students [3–5]. The extended leave of absence along with minimal clinical exposure during PhD training promotes a natural loss of clinical skills and knowledge, which presents unique challenges in meeting the demands of clinical training. MD-PhD students frequently report feeling anxious, isolated, and less prepared than their MD counterparts for clinical training [3–5]. These feelings are justified; in addition to subjective thoughts of clinical inadequacy, there is evidence that MD-PhD students score significantly lower than MD students on standardized clinical examinations [4]. Another challenge faced by MD-PhD students is the accelerated training timeline, which can limit opportunities to encounter and observe various medical specialities. This poses a unique challenge for MD-PhD students in choosing a specialty and preparing for residency. In order to combat these issues, a recent survey suggested that greater than 80% of dual-degree students would be willing to devote 0.5–1.0 hours per week to continuing clinical education (CCE) course during their PhD training [5].

Clinical courses prior to the transition to clinical training have been utilized to help offset decline of clinical skills and knowledge during PhD training [4,7–9]. Limited published data suggest these courses give students more confidence in conducting history and physical examinations and may improve performance on clerkships and observed clinical skills evaluations [5,8,9]. However, these courses vary in duration, intensity, content, extent of student participation, and metrics of success (Table 1), making it difficult to compare course efficacy. As such, there is no consensus on the ideal format for such a course.

**Table 1.** Clinical education courses for MD-PhD students during PhD training

Institution	Duration	Components	Evaluation measures	Results
UAB	All PhD years (required)	(a) Clinical encounters: observed H&P with residents, faculty-supervised volunteering at student-run free clinic, (b) shadowing clinical faculty, (c) Clinical Knowledge Sessions: case and hospital rounds simulations, NEJM case conferences, medicine and pediatric morning reports, Kaizen question sessions, (d) optional: clinical skills workshops, shadowing an inpatient medicine clerkship team	Structured student survey	Course components were helpful to maintain clinical skills and knowledge, gain exposure to clinical specialties during PhD training, and prepare them for responsibilities during clinical training (clerkships)
Vanderbilt [9]	All PhD years (required)	Student group paired with clinical preceptor and students conduct H&Ps then meet monthly with preceptor. Topics discussed at meetings: (a) G1 Year: review H&P, oral presentations, and differential diagnoses; (b) G2/G3 Years: improve and refine H&P and oral presentation skills, review differential diagnoses, clinical approach introduction; (c) G4/G5 Years: refine H&P and oral presentation skills, review differential diagnoses and clinical procedures, and broaden clinical approach	Clerkship grades	No significant differences in clerkship grades between MD and MD-PhD students (*P* = 0.27)
UCSD [4]	6 weeks prior to start of clinical training, one half-day per week (optional)	Week 1: students work in pairs and practice physical examinations	Student feedback (format unknown)	Efforts were a good use of time, and program should be offered to future students
		Week 2: OSCE participation	OSCE scores	
		Weeks 3-6: one H&P weekly observed by faculty (one inpatient and three outpatient)		MD-PhD students had significantly lower OSCE scores than MS2 MD students (*P* = 0.01), notably the cardiovascular and pulmonary exams (*P* = 0.05)
Icahn School of Medicine at Mount Sinai [7,8]	9 weeks prior to the start of clinical training (required)	Week 1: one day Clinical Basics Orientation session (review H&P and practice with standardized patients)	Student survey (format unknown)	MD-PhD students taking the course reported increased confidence in H&P skills, interpreting laboratory data, generating differential diagnoses, formulating assessments and plans, writing notes, and understanding the role of a medical student during clinical training
			OSCE scores	
			Clerkship performance	
		Week 2–9: once weekly 4-hour morning sessions: H&Ps that are supervised by a physician preceptor intermittently and case presentations followed by case discussions (3 weeks), inpatient medicine team shadowing (1 week), independent H&Ps observed by inpatient attending (3 weeks), and OSCE (1 week)		
				No significant differences in OSCE scores between MS2 MD and MD-PhD students (*P* > 0.05)
				A higher proportion of MD-PhD students received clerkship Honors after course implementation

G1 = First-year graduate research (PhD) training; H&P = history and physical examination; MS2 = second-year medical student; NEJM = New England Journal of Medicine; OSCE = Objective Structured Clinical Examination; UAB = University of Alabama at Birmingham; UCSD = University of California, San Diego.

Longitudinal clinical courses taken by MD-PhD students during all years of PhD training offer the potential to maintain and likely build, knowledge and skills rather than re-learn them. In recent years, many MD/PhD programs have acknowledged the importance of these types of courses, but published data and discussion on their implementation remain limited. Currently, only one such course has been described in the literature, which focuses primarily on clinical skills [9]. In contrast, the University of Alabama at Birmingham (UAB) MD-PhD program developed a longitudinal CCE course taken in all PhD years that encompases clinical skills and knowledge activities as well as physician shadowing and represents the most comprehensive clinical course for MD-PhD students in their PhD years reported to date. We also designed a structured student survey of the course to be completed at the end of each semester in order to evaluate the effectiveness of course components. Here, we describe the CCE course and post-course survey findings.

## Materials and Methods

### Study Subjects

The UAB MD-PhD program is an NIH NIGMS-funded Medical Scientist Training Program (MSTP). The core curriculum structure consists of integrated medical and graduate school courses during the initial 2 years, followed by a multi-year PhD training phase, and a final clinical training phase. With the exception of the 1-month family medicine clerkship in the final months of their MS2 year, MD/PhD trainees at UAB do not start clinical rotations until the final clinical training phase.

### CCE Course Design

**Figure 1.**
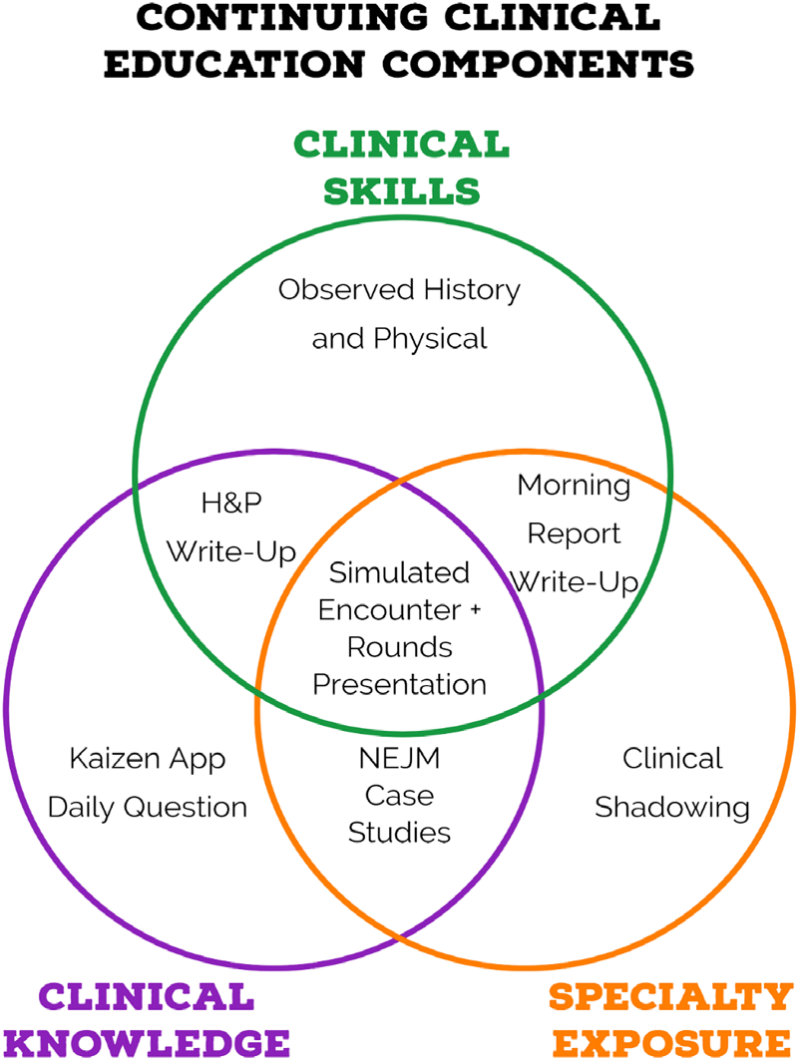
Course components. Venn diagram of all course activities that can be completed for credit in the UAB MSTP continuing education course. Each activity encompasses at least one of the three course goals of maintaining clinical skills, maintaining clinical knowledge, and exposing the student to different specialties they may be interested in pursuing residency training in.

The CCE course, designed by UAB MSTP leadership and students, was initiated in 2012 and underwent a major revision in 2014 based on student and faculty feedback. The course features a three-component curriculum focusing on: (1) maintaining clinical skills, (2) maintaining clinical knowledge, and (3) exposing students to different specialty areas. To achieve these goals, an array of activities have been developed and added to over time. Each activity focuses on at least one of the three components, with several activities overlapping multiple components (Fig. 1). Students are required to complete at least one observed clinical encounter, one shadowing experience, two activities in the clinical knowledge component, and two additional activities of the student’s choice per semester (Table 2). During the COVID-19 pandemic when in-person clinical activities were limited, modifications were made such that students could complete any six activities of their choice. Specifically, students were unable to enter clinical settings to complete observed histories and physicals and were barred from most shadowing opportunities. Students spend about 12–15 hours each semester completing course requirements, and those in their final PhD year are encouraged to complete additional optional clinical activities.

**Table 2.** University of Alabama at Birmingham (UAB) Medical Scientist Training Program (MSTP) continuing clinical education course curriculum

Category	Activity	Assignment	Minimum # required per semester
Clinical Encounter	Observed history and physical examination with medicine or pediatric resident	Patient write-up	1
	Volunteer at student-run free health clinic		
Shadowing	Shadow clinical faculty	Evaluation form	1
Clinical Knowledge	NEJM case conference	Attendance	2
	Medicine or pediatrics morning report	Case summary form	
	Simulation session	Attendance	
	Kaizen education session	Complete minimum of 25 questions	
Choose Your Own Activity	Any of the above activities		2

NEJM = New England Journal of Medicine.

The course is managed by a MSTP student-led oversight committee and supervised by a faculty course director. Students in the program manage scheduling faculty speakers, simulation labs, and organizing questions and incentives to complete daily clinical vignette style questions. As students play an integral role in syllabus development and are able to first-hand experience how course requirements shape their return to clinic, the course is continuously evolving around students’ goals.

To evaluate MSTP students’ perception of the course effectiveness and obtain suggestions for improvement, students complete an anonymous survey at the end of each semester. Key survey questions are listed in Table 3, which have been modified in recent years to address additional course components.

**Table 3.** University of Alabama at Birmingham (UAB) Medical Scientist Training Program (MSTP) continuing clinical education course evaluation survey

**Please indicate whether you (a) Strongly agree, (b) Agree, (c) Disagree, (d) Strongly disagree with, or (e) Cannot evaluate the following statements:**
The resident supervised clinical encounter was a useful way for me to improve and maintain my clinical skills
The EAB clinical encounter was a useful way for me to improve and maintain my clinical skills
I learned about the day to day life in a particular specialty from my shadowing experience
The shadowing experience helped me prepare for my responsibilities as an MS3
The shadowing experience helped me identify a potential specialty for residency
I found the NEJM cases to be helpful in retaining and/or improving my clinical knowledge
I found my UAB SIM lab experience to be a helpful way to retain and/or improve my clinical knowledge
I found my Samford SIM lab experience to be a helpful way to retain and/or improve my clinical knowledge
I found morning report to be a helpful way to retain and/or improve my clinical knowledge
**Please indicate the degree to which this course met its objectives: (a) Satisfactorily met objective, (b) Partially met objective, (c) Did not meet objective**
Help students maintain and mature the clinical skills acquired during the first two years of medical school
Allow students to gain exposure to different clinical specialties
Help students maintain and mature clinical knowledge during the first two years of medical school
**Please indicate the amount of time spent on this course: (a) Way too much, (b) Too much, (c) About right, (d) Too little, (e) Way too little**
The time I spend on this course is:
**Overall, how can we improve the course?: open ended responses recorded.**

EAB = Equal Access Birmingham clinic; NEJM = *New England Journal of Medicine*; SIM = simulation.

### Observed clinical encounters

Observed clinical encounters are designed to maintain a student’s ability to take a history and complete a thorough physical examination. For this activity, students are required to complete a history and physical (H&P) on a real patient while being observed by a resident or attending physician. Students may complete this H&P either on an admitted hospital medicine patient with a resident or at UAB’s student-run free clinic with an attending. Verbal feedback is provided to the student immediately following the encounter. Students are then required to write up the H&P encounter, including an assessment and plan. Students are encouraged to give a formal oral presentation and show their completed patient write-ups to their observer for additional feedback, though this is not a requirement for credit.

#### Shadowing experiences

Shadowing experiences are designed to expose students to multiple clinical specialties, with the goal of guiding future residency decisions. Students are required to shadow a clinical faculty member in any specialty at least once each semester and submit an evaluation form to receive credit. Students shadow faculty in a variety of clinical settings including outpatient clinic, inpatient hospital rounds, during surgeries or procedures, and other clinical areas, typically shadowing multiple faculty from different specialties throughout their PhD training

#### New England Journal of Medicine (NEJM) case conferences

In the NEJM case conferences, clinical faculty lead students through an interactive clinical case discussion, including differential diagnosis, evaluation, treatment, and follow-up for a patient scenario [10]. Discussion is based on one of the NEJM Interactive Medical Cases with additional teaching pearls provided by the specialty moderator. These hour-long sessions are offered four times each semester, and credit is given for attendance. A faculty member of the most relevant field of study serves as the moderator and presenter for discussion, that is, an infectious disease attending will moderate a case focusing on the differential and work-up for cases involving an infectious disease. This rotating moderator role allows for students not only to hear from an expert in the specific area but also allows for students to network with faculty from unique specialty areas.

#### Medicine and pediatric morning reports

Medicine and pediatric morning reports occur daily at UAB Hospital and Children’s of Alabama, respectively, and consist of a clinical faculty member leading an interactive discussion of a clinical case presented by a resident. Students must submit a morning report summary form to receive credit, requiring them to distill the presentation and discussion into pertinent facts and findings of the patient case, evaluation, diagnosis, and management.

#### Simulation sessions

Simulation sessions allow students to apply clinical knowledge in a controlled practice setting. Two types of simulations are offered. The first is a case-based simulation, in which a clinical faculty member facilitates a case using a manikin and a team of students work together to arrive at a diagnosis and treatment plan. This is followed by the faculty member debriefing the students on relevant clinical aspects of the case and their performance. The second simulation, a hospital rounds simulation, was introduced in fall 2017 and is conducted in collaboration with Samford University McWhorter School of Pharmacy. During this simulation, UAB MSTP students and Samford pharmacy students pre-round on two standardized patients and then present the cases to a facilitator (a UAB or Samford faculty member or UAB fellow or resident) on simulated rounds and then repeat. This is followed by a debriefing session in which they review the clinical aspects of the cases and discuss different aspects of hospital rounds. Credit for simulations is awarded based on attendance. Both simulation experiences serve as a way to ease students into their future MS3 roles and allows them to practice H&Ps and presentation skills in standardized, low-stakes environments, while simultaneously exposing students to faculty moderators from multiple specialty areas.

#### Kaizen education platform daily question app

The UAB-developed Kaizen education platform is an innovative online gaming platform adapted by the UAB MSTP for the CCE course in fall 2018 as a way to continue evidence-based spaced repetition learning to enhance clinical knowledge retention [11,12]. The MD-PhD-specific Kaizen design consists of two 4-week rounds of questions. Each question is followed by detailed explanations, thereby providing real-time feedback. For each 4-week round, a new clinical vignette multiple-choice question is released daily, testing students’ clinical knowledge and preparing them for the USMLE Step 2 CK examination. Students must answer at least 25 questions in each round to receive credit.

#### Optional clinical activities

Optional clinical activities available to students in their final year of PhD training include clinical skills workshops and the opportunity to shadow an Internal Medicine inpatient team. The workshops provide MSTP students with essential clinical skills, including basic approaches to ordering laboratory tests, reading electrocardiogram (EKGs) and imaging studies, and hands-on training in suturing, intubation, and placing intravenous lines. Each workshop is led by clinical faculty and attended by groups of 10–12 students. MSTP students may also shadow an Internal Medicine inpatient team, consisting of third and fourth-year medical students, Internal Medicine residents, and an Internal Medicine attending, to gain an understanding of the responsibilities and expectations for their upcoming clinical clerkships. A “bootcamp” style 1-day session was trialed in 2022 for students returning to MS3 and is being further refined

### Data Analyses

Analyses were conducted on SPSS (version 27.0, Armonk, NY: IBM Corp.).

#### General survey analyses

Study survey question responses are presented as number and percentages of responses during the study period from 2014 to 2022 and reflect the combination of “strongly agree” and “agree” responses or combination of “satisfactorily” and “partially” met objective. A final question on the survey asks students to report, on a 1–4 scale, whether they feel confident in their ability to return to clinic, with “1” indicating they are “Not at all ready” and have “serious concerns about [their] skill level” while “4” indicates they are “Very ready” and “confident in [their] abilities.” To complete the survey, not all responses are required – even for activities the respondent did complete. Therefore, the overall respondents for each question are presented in results.

#### COVID-19 semester analyses

Six of the 17 semesters were designated as “COVID-19” semesters (spring 2020–fall 2022) as fall 2022 saw a gradualf return of in-person clinical activity options. Survey responses of whether the CCE course met objectives before and after COVID-19 were compared with Chi-square analyses. Average clinical confidence scores were compared between semesters before and after COVID-19 using Mann–Whitney U analysis and Spearman’s correlations.

## Results

### General Survey Analysis

There were 597 surveys completed by MD-PhD students enrolled in the CCE course between 2014 and 2022. As students complete an average of eight CCE course semesters, students are counted multiple times and will be referred to as “survey respondents” instead of “students.”

Regarding observed clinical encounters, most survey respondents reported clinical encounters were useful for maintaining and improving clinical skills (494/537, 92%). Of the 41% of respondents who requested formalized feedback on their written H&P from this encounter, 98% of them found that feedback is constructive and beneficial.

In terms of shadowing experiences, the majority reported that shadowing prepared them for their responsibilities during clinical training (301/397, 76%). Additionally, most respondents reported that shadowing helped them learn about the day-to-day life in a particular specialty (391/405, 97%) and identify a potential specialty for residency (344/397, 87%).

The NEJM case conference was the most widely completed course activity and most (559/568, 98%) found it to be a helpful way to retain and/or improve clinical knowledge. Regarding other clinical knowledge activities offered, respondents reported the case-based simulation, hospital rounds simulation, and morning report to be a helpful way to retain and/or improve clinical knowledge (111/114, 97%; 70/71, 99%; and 297/305, 97%, respectively). Kaizen was shown to be an effective learning platform from our recent published study [11].

**Figure 2.**
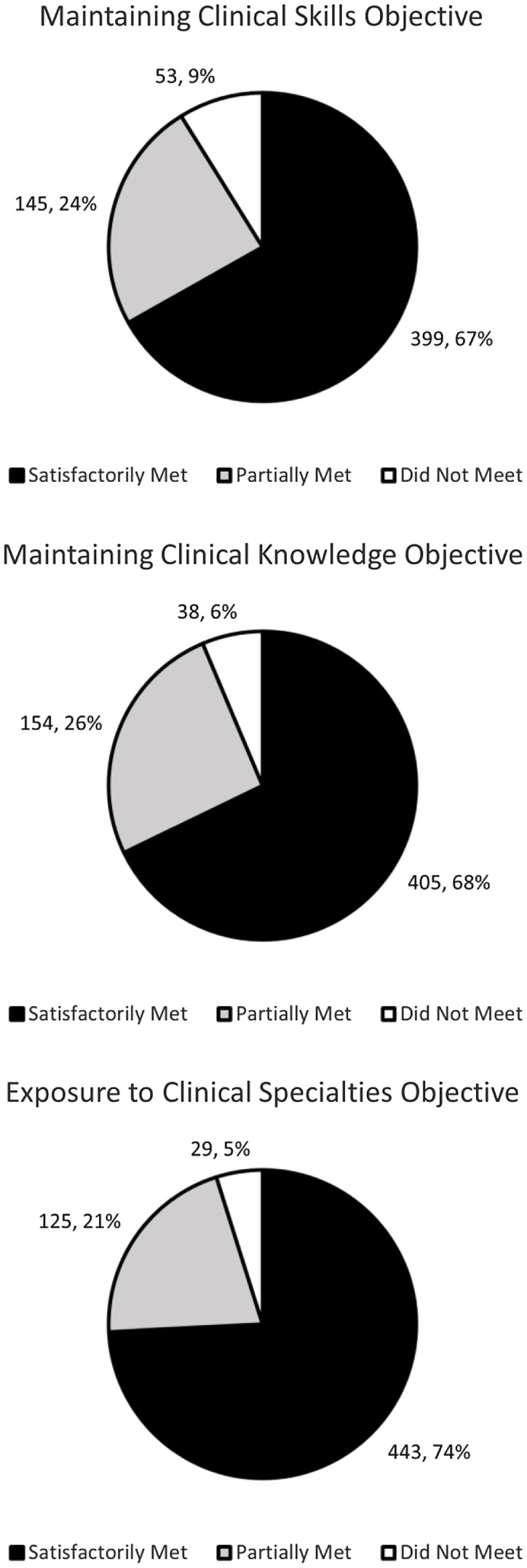
Course goals. Pie charts depicting the proportion of students believing each course objective was satisfactorily met, partially met, or not met.

The proportions of respondents believing the CCE course satisfactorily met, partially met, or did not met each of the three course objectives (maintaining clinical skills, exposing students to different specialties, and maintaining clinical knowledge) can be seen in Figure 2. Overall, 544/597 (91%) of respondents believed the CCE course either satisfactorily or partially met the clinical skills objective, 568/597 (95%) believed it either satisfactorily or partially met the exposure objective, and 559/597 (94%) believed it either satisfactorily or partially met the clinical knowledge objective.

### COVID-19 Semester Analysis

A higher proportion of respondents reported the course met its goal of maintaining clinical knowledge during the COVID-19 semesters than prior to the pandemic (222/229 [96.9%] vs. 337/368 [91.5%], *p* = 0.01). There was no significant difference in the proportion of respondents who believed the CCE course met versus did not meet its objective for maintaining clinical skills (*p* = 0.49) or exposing students to different specialties (*p* = 0.36). There was a significant difference between clinical readiness scores between pre-COVID-19 and COVID-19 semesters (*p* = 0.03), with lower readiness scores during the COVID-19 semesters.

**Figure 3.**
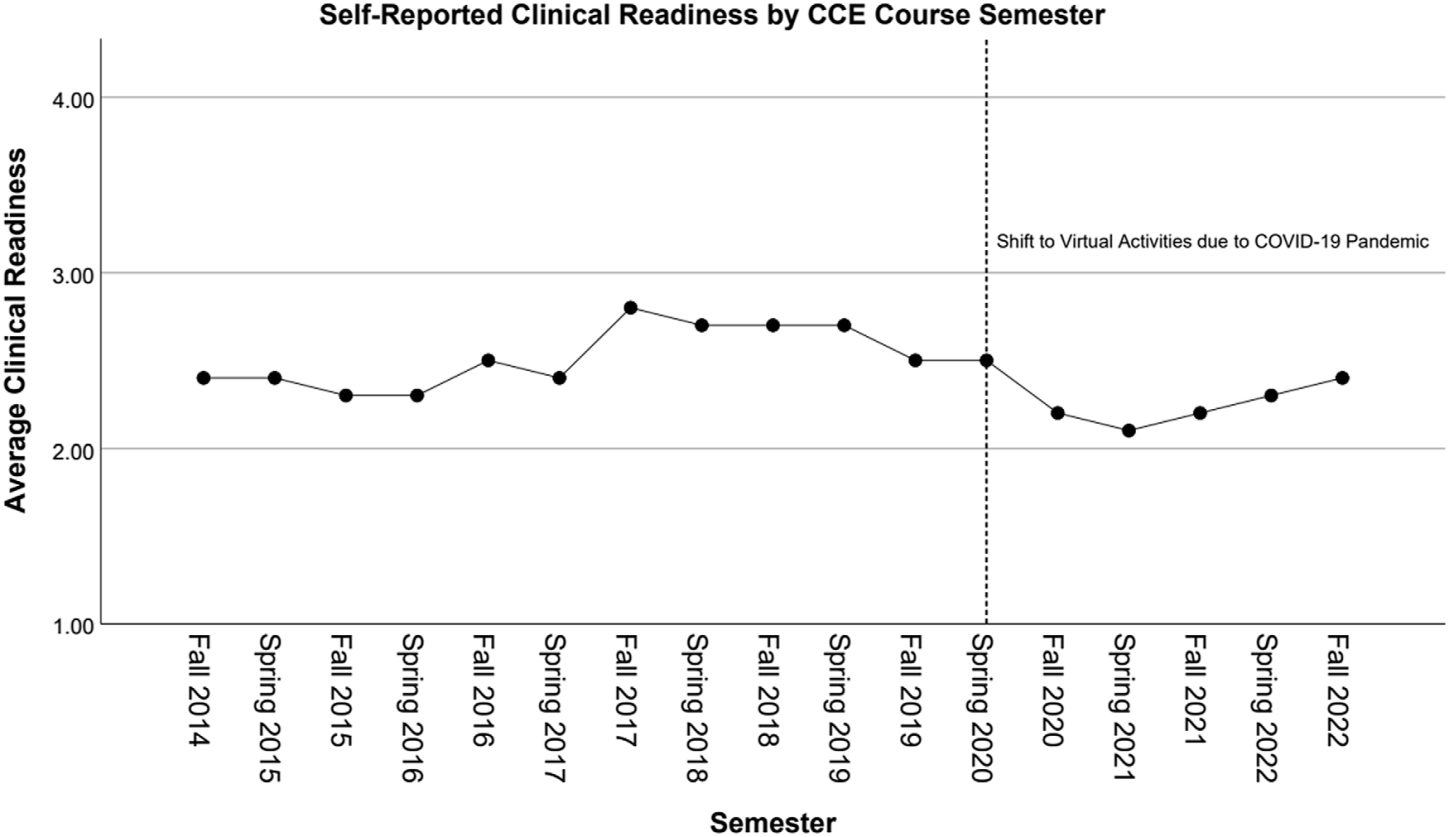
Clinical readiness over time. Graph depicting the average clinical readiness reported by students each semester, ranging from 1 to 4 with 1 being “not at all ready” and 4 being “very ready.” The start of the COVID-19 pandemic is indicated by the dashed line and was associated with a significant drop in clinical readiness that has slowly begun to resolve as more clinical opportunities have reopened to students. Fall 2022 saw the complete return to normal in-person activities seen prior to Spring 2020. CCE = continuing clinical education.

Over the course’s evolution, including all semesters, there was no significant change in students’ confidence in their readiness to return to clinic (Spearman’s *r* = −0.24, *p* = 0.35). Prior to the spring 2020 semester, there was a significant correlation with increasing readiness confidence (Spearman’s *r* = 0.69, *p* = 0.02). During the six COVID-19 semesters, there was no correlation with increasing readiness (Spearman’s *r* = 0.03, *p* = 0.96). Average readiness responses can be seen graphed over time in Figure 3.

## Discussion

We designed and implemented a comprehensive clinical education course for UAB MSTP students during their PhD training phase that maintains clinical knowledge and skills prior to re-entry to clinical training. With a student time commitment of about 12–15 hours each semester, our course provides sufficient time and flexibility to participate in activities while not impairing PhD training and dissertation completion. The course survey results indicated that students valued the continuous clinical learning approach and opportunities to explore medical specialties to guide residency decisions.

Overall, over 91% of students felt each of the three course objectives (maintaining clinical skills, exposing students to different specialties, and maintaining clinical knowledge) were at least partially met each semester. Students generally felt that structured activities that integrated multiple course objectives at once were most helpful. Specifically, almost every student (97%–100%) who completed simulation experiences, NEJM case study discussions, and/or morning reports stated the activity they completed was helpful for maintaining clinical skills and knowledge. Activities with less structure, such as observed resident H&Ps and shadowing experiences, were still viewed as helpful in preparation for clinic (92% and 75%, respectively), though not to the same extent. This may be due to difficulties some students had organizing sessions with time-constrained residents and lack of ability to contribute clinically in a shadowing setting.

It should be noted that observed H&P sessions and shadowing activities were required components of the course except for early COVID-19 pandemic semesters. While each student must complete the same standardized number of activities, the remainder can be chosen by the student themselves. Therefore, it is possible that individuals who completed simulation labs, morning reports, and NEJM cases specifically sought out those activities as their choices because they find them enjoyable and/or useful. This may have affected our findings that students found the observed H&P sessions and shadowing activities the least useful. In addition, while shadowing experiences may not focus on improving a student’s personal knowledge or clinical skills, the exposure and networking it provides are important for students exploring different clinical fields early for tailoring future MS3/4 course scheduling.

As the course has evolved and added new activities, such as the hospital rounds simulation and Kaizen educational game, there was an increase in the average student’s confidence to return to clinic from its implementation in 2014 to later iterations in 2019. However, the COVID-19 pandemic saw a significant drop in reported clinical readiness, despite students continuing to believe the CCE course was meeting each of its objectives. This is likely due to a multitude of factors affecting students, including external factors relating to the COVID-19 pandemic unrelated to medical education, but a major factor is likely the lack of exposure to in-person clinical activities as most other course activities were able to move to a virtual format and continue. This finding underlies the importance of exposing students during their PhD phases to regular clinical activities to build confidence in returning to medical school, whether or not a part of a larger course.

Despite its noteworthy strengths, our course has limitations. Our findings are from a single MD-PhD program; thus, it is unknown if findings would be generalizable to other MD-PhD programs implementing a similar CCE course. We hope that this description of our MSTP and CCE course as well as the provided study survey questions might help other MD-PhD programs in implementation and evaluation of a similar course. Importantly, this course requires considerable coordination of activities by students of our program as well as support from multiple residents and faculty, which may not be possible in all MD-PhD programs. While this study reports outcome data from student surveys, we had also intended to evaluate the course’s impact on another objective measure, National Board of Medical Examiners shelf exam scores; however, changes in required curriculum and shelf exam scoring norms between the time periods impacted comparability, and therefore these measures were not analyzed.

All survey data collected during this study focused on students still in their PhD phase. Future directions for this work would include reaching out to trainees after making their transition back to clinic to identify how accurate they were in their perception of their clinical readiness. For example, do students who feel more prepared feel like they perform better on their clinical rotations and do shelf exam/Step 2 scores reflect this? Once in their clinical years, looking back what experiences were most helpful from the CCE course? There are many important adjustments to our course by taking these post-transition opinions into consideration.

## Conclusions

The UAB MSTP implemented a comprehensive clinical education course spanning all PhD training years to maintain clinical skills and knowledge of MD-PhD students during their PhD training to better prepare for the transition to clinical clerkships. While initial implementation of the course led to a gradual increase in students’ clinical confidence, the COVID-19 pandemic prevented in-person clinical course activities and was associated with a significant drop in confidence that has itself gradually improved. Positive student survey feedback and improved clinical confidence suggest the CCE course is a useful and effective approach to maintain clinical knowledge during research training.

## Data Availability

The datasets generated during and/or analyzed during the current study are available from the corresponding author on reasonable request.
